# Appetite and its Regulation: Are there Palatable Interventions for Heart Failure?

**DOI:** 10.1007/s11897-023-00637-7

**Published:** 2023-12-22

**Authors:** Matthew M. Y. Lee, Michael E. J. Lean, Naveed Sattar, Mark C. Petrie

**Affiliations:** 1 School of Cardiovascular and Metabolic Health, University of Glasgow, Glasgow, UK; 2 Human Nutrition, School of Medicine, Dentistry & Nursing, College of Medical, Veterinary & Life Sciences, University of Glasgow, Glasgow, UK

**Keywords:** Appetite, Body weight, Diet, Heart failure, Obesity, Weight-loss drug

## Abstract

**Purpose of Review:**

Obesity is a major driver of heart failure (HF) incidence, and aggravates its pathophysiology. We summarized key reported and ongoing randomized clinical trials of appetite regulation and/or dietary energy restriction in individuals with HF.

**Recent Findings:**

Weight loss can be achieved by structured supervised diet programs with behavioural change, medications, or surgery. The new glucagon-like peptide-1 receptor agonists alone or in combination with other agents (e.g., glucose-dependent insulinotropic polypeptide and glucagon receptor agonists or amylin analogues) potently and sustainably reduce appetite, and, taken together with dietary advice, can produce substantial, life-changing, weight loss approaching that achieved by surgery. To date, data from the STEP-HFpEF trial show meaningful improvements in health status (Kansas City Cardiomyopathy Questionnaire).

**Summary:**

Effective weight management could relieve several drivers of HF, to complement the existing treatments for HF with both reduced and preserved ejection fraction. Further trials of weight loss interventions will provide more definitive evidence to understand their effects on clinical events in patients with HF.

## Obesity and Heart Failure (HF)

Obesity increases metabolic rate and demand on the cardiovascular and respiratory systems, is a major driver of HF incidence, and aggravates its pathophysiology: the heart, like any machine, will fail if the systemic demand or load is too great. Combined, HF and obesity cause an especially poor quality of life, with more HF hospitalizations and cardiovascular deaths [1, 2].

## Appetite Regulation

Appetite, defined as a desire for food, is regulated centrally by the hypothalamus, and by several hormones secreted peripherally, some in response to dietary intake.

## Weight Loss

While unintentional weight loss usually indicates disease and poor prognosis, modifying appetite to induce negative caloric (energy) balance generates *intentional* weight loss, appears to be an efficacious treatment option for people with HF and obesity, to treat or alleviate HF and comorbidities [3–5]. Weight loss is possible with structured supervised diet programs with behavioural change, but only sustainable if appetite can be overcome or altered, by professional and lay support, changes in food choices, medications, or surgery [6]. Whilst bariatric surgery usually generates major weight loss, it is a more challenging option in the presence of HF, and late weight regain and side-effects (e.g. dumping syndrome, vitamin and mineral deficiencies, need for re-operation or reversal) are common problems. Evidence-based diet programmes including nutritional formula ‘total diet replacement’ for the weight loss phase can reliably produce weight loss > 10 kg at 12 months [6]. The new GLP-1 receptor agonists, GIP and glucagon receptor agonists, potently and sustainably reduce appetite, and, taken together with dietary advice, can produce substantial, life-changing, weight loss approaching that achieved by surgery [7, 8]. Are these strategies ‘palatable’ to individuals with HF and to healthcare systems? We recently suggested targeting more conditions earlier with weight loss may reap multiple gains (including reducing multimorbidity) beyond currently recommended treatment options [3].

## Reported and Ongoing Clinical Trials (Fig. 1)

**Fig. 1:**
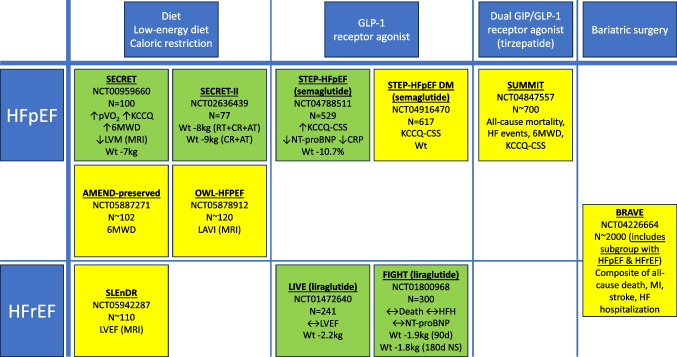
Key randomized trials of appetite regulation in HF (key outcomes; green = completed; yellow = ongoing; between-group differences shown except for SECRET-II where within-group differences shown). Abbreviations: 6MWD, 6-min walk distance; AT, aerobic exercise training; CR, caloric restriction; CRP, C-reactive protein; GIP, glucose-dependent insulinotropic polypeptide; GLP-1, glucagon-like peptide-1; HF, heart failure; HFH, heart failure hospitalization; HFpEF, heart failure with preserved ejection fraction; HFrEF, heart failure with reduced ejection fraction; KCCQ-CSS, Kansas City Cardiomyopathy Questionnaire Clinical Summary Score; LAVI, left atrial volume index; LVEF, left ventricular ejection fraction; LVM, left ventricular mass; MRI, magnetic resonance imaging; NS, not significant; NT-proBNP, N-terminal pro-B-type natriuretic peptide; pVO_2_, peak oxygen uptake; RT, resistance training; wt, body weight

In HF with preserved ejection fraction (HFpEF), the largest diet-driven randomised trial (SECRET; *n* = 100) of a dietary intervention (caloric restriction (provision of all foods in a university setting)) resulted in 7 kg weight loss and improved 6-min walk distance, peak oxygen uptake, health status, and regression in left ventricular mass, compared to those who did not undergo the diet intervention [4]. Based on this one trial, caloric restriction is listed as a treatment option in international guidelines and expert consensus documents [9, 10]. Other diet trials in HFpEF were smaller, non-randomised, or achieved only modest weight loss (< 10 kg) [11, 12]. In STEP-HFpEF (*n* = 529) the GLP-1 receptor agonist semaglutide reduced weight by 10.7 %, improved health status, reduced a hierarchical composite endpoint, improved exercise function, and reduced inflammation and NT-proBNP [5]. In HF with reduced ejection fraction (HFrEF), dietary studies have been small or non-randomised [13]. In HFrEF, two trials in non-obese populations of the GLP-1 receptor agonist liraglutide achieved trivial weight loss (~ 2 kg); there were very small numbers of clinical endpoints in these trials, rendering unclear conclusions [14, 15]. There are no randomized trials of bariatric surgery in HFpEF or HFrEF, only observational evidence that supports a potential benefit from intentional weight loss [16]. The ongoing BRAVE open-label randomized controlled trial (NCT04226664) is assessing bariatric surgery versus medical weight management and will recruit ~ 2000 patients with high-risk cardiovascular disease (including, importantly, a subgroup (interim data *n* = 17/49 (35%)) with symptomatic HF) [17] (Fig. 1).

## Future Directions

From first principles, earlier, effective weight management with substantial weight loss should relieve several drivers of HF, to complement the existing treatments for HFrEF and potentially provide a vital effective intervention for HFpEF. Adequately powered outcome trials are needed to provide definitive evidence, to understand the effect of *intentional* weight loss on clinical events, and mechanisms of benefit. The value of combining therapies must be assessed: GLP-1/GIP/glucagon receptor agonists appear promising adjunctive treatments that warrant definitive trials, and it remains possible that some effects of these medications may be independent of weight loss. Challenges include long-term weight-loss maintenance, programme accessibility, flexibility and adherence, and costs. Hippocrates reminded us: “*If we could give every individual the right amount of nourishment and exercise, not too little and not too much, we would have found the safest way to health*.” [18] Healthcare systems need to target weight loss much more actively for societal and patient gains [3].

## Data Availability

All supporting data are available within the cited references. No new data were generated in support of this review.
